# The defensive benefit and flower number cost of selenium accumulation in *Brassica juncea*

**DOI:** 10.1093/aobpla/plz053

**Published:** 2019-08-17

**Authors:** Janet C Steven, Alexander Culver

**Affiliations:** 1 Department of Organismal and Environmental Biology, Christopher Newport University, Newport News, VA, USA; 2 New Horizons Governor’s School for Science and Technology Hampton, VA, USA

**Keywords:** Accumulation, *Brassica juncea*, elemental defence, herbivory, hyperaccumulation, *Pieris rapae*, selenium

## Abstract

Some plant species accumulate selenium in their tissues in quantities far above soil concentrations, and experiments demonstrate that selenium can serve as a defence against herbivores and pathogens. However, selenium may also cause oxidative stress and reduce growth in plants. We measured growth, selenium accumulation and herbivory in four varieties of the selenium accumulator *Brassica juncea* to investigate the cost of accumulation as well as its benefit in reducing herbivory. We measured selenium levels, plant size and flower number in four varieties of *B. juncea* watered with sodium selenate or treated as controls. We also conducted no-choice herbivory trials on leaves from both treatments with the specialist herbivore *Pieris rapae*. The selenate treatment slightly increased leaf number over the control, but tissue concentrations of selenium and flower number were negatively correlated in some varieties. In herbivory trials, leaves from the plants in the selenate treatment lost less leaf tissue, and the majority of larvae given leaves from selenate-treated plants ate very little leaf tissue at all. In the variety with the highest selenium accumulation, leaves from selenate-treated plants that showed reduced flower production also experienced less herbivory in feeding trials. The protective advantage of greater selenium accumulation may be offset by negative effects on reproduction, and the relatively low level of selenium accumulation in this species as compared to more extreme hyperaccumulators could reflect the minimum level necessary to enhance protection from herbivory.

## Teaser

Selenium is an essential element for animal health, but it can be toxic at high concentrations. Plants are known to exploit this by storing high levels of selenium in their leaves and poisoning their animal enemies. We found that brown mustard plants that absorbed selenium from the soil and accumulated it in their leaves were in fact eaten less by a moth larva. However, at high concentrations of selenium plants also made fewer flowers. Accumulating selenium protects the plants from herbivores, but it also may come at a cost in reproduction.

## Introduction

Selenium is not known to be an essential element for plants, but it is associated with enhanced plant growth and protection from abiotic stress at low concentrations (Pilon-Smits *et al.* 2009; Statwick *et al.* 2016) and protection from herbivory and pathogens at a range of concentrations (reviewed in Schiavon and Pilon-Smits 2017). However, at higher concentrations selenium can be toxic to plants (Brown and Schrift 1982; Gupta and Gupta 2017). While many plants seem to take up and metabolize selenium inadvertently through sulfur acquisition pathways (Pilon-Smits 2015), some species have specialized mechanisms to take up and sequester selenium and can reach concentrations of tissue selenium above 1000 μg g^−1^; these species commonly occur on seleniferous soils and are labelled as hyperaccumulators (Rascio and Navari-Izzo 2011; White 2016; Schiavon and Pilon-Smits 2017). Selenium accumulation in concentrations below 1000 μg g^−1^ also occurs in species that do not colonize soils high in selenium and appear to lack specialized adaptations for uptake and storage of selenium (Brown and Shrift 1982; Statwick *et al.* 2016). Plants in this category are variously called secondary Se accumulators (Brown and Shrift 1982), Se indicators (Rosenfeld and Beath 1964) or simply accumulators (Hanson *et al.* 2003). While hyperaccumulators show no evidence of toxicity to selenium, increased selenium content in accumulators may reduce growth (Bañuelos *et al.* 1997). In this study, we explored the lower limits at which selenium concentration serves as a defence against herbivory for *Brassica juncea*, an accumulator, and whether these concentrations showed a cost to plant growth.

The elemental defence hypothesis states that hyperaccumulation of certain elements to levels many times above soil concentrations can defend plants against insect herbivores (Boyd and Martens 1992; Boyd 2007; Vesk and Reichman 2009). Hyperaccumulation of selenium has evolved multiple times in angiosperms and selenium hyperaccumulators are present in 21 plant families, suggesting that it has adaptive benefits to the plant (Cappa and Pilon-Smits 2014). Laboratory trials have shown selenium to be an effective defence against Orthoptera (Freeman *et al* 2007), aphids (Hanson *et al.* 2004), prairie dogs (Quinn *et al.* 2008), Lepidoptera larvae and fungal pathogens (Hanson *et al.* 2003). Selenium hyperaccumulators also experienced lower arthropod loads than non-accumulators in a natural habitat (Galeas *et al.* 2008). In addition, there is some evidence that the lower concentrations of selenium found in accumulators may also provide a defence against herbivores. Beet armyworm larvae experienced an LC_50_ for selenium in artificial diet below 50 μg g^−1^, indicating toxicity at concentrations far below the level that defines species as hyperaccumulators (Trumble *et al.* 1998). A species of *Astragalus* with broad habitat requirements and no particular adaptations for selenium uptake showed a defence against herbivores when grown in selenium-rich soil (Statwick *et al.* 2016). In addition, concentrations of selenium as low as 10 μg g^−1^ decreased aphid herbivory on *B. juncea* (Hanson *et al.* 2004) and levels as low as 38 μg g^−1^ deterred prairie dog herbivory in *Stanleya pinnata*. The elemental defence hypothesis may explain the lower concentrations of selenium found in accumulators as well as the high levels found in hyperaccumulators.

Although high selenium concentrations are potentially beneficial to plants, increased concentrations in accumulators may also damage plant tissues. Selenium can induce oxidative stress and take the place of sulfur in proteins, resulting in protein malformation (Brown and Shrift 1982; Gupta and Gupta 2017). Simultaneous positive and negative effects of selenium have been reported in radish; selenium accumulation did decrease herbivory but it also caused reduced biomass and increased fruit abortion (Hladun *et al.* 2013). Accumulation of selenium in the accumulator *B. juncea* impairs pollen germination (Quinn *et al.* 2011), and high concentrations of selenium or metals in pollen and nectar can reduce pollinator visitation and influence the identity of pollinators (Meindl and Ashman 2014). Selenium concentrations in accumulators may reflect an equilibrium point between the benefits of deterring herbivores and the damage and growth reductions caused by excess selenium (Boyd 2012).

To identify the relative positive and negative impacts of selenium concentrations on herbivore defence and plant growth, we measured selenium concentration and growth in four varieties of *B. juncea* and conducted non-choice feeding trials with the specialist herbivore *Pieris rapae*. *Brassica juncea* is capable of absorbing selenate rapidly from the soil and accumulating selenium in above-ground tissues (Bañuelos *et al.* 1997), and the presence of selenium in *B. juncea* tissues reduces herbivory by *P. rapae* (Hanson *et al.* 2003). However, *B. juncea* is known to lack the adaptations to extremely high selenium accumulation present in selenium hyperaccumulators, including preferential absorption of selenate over sulfur and storage in young leaves (Feist and Parker 2001; Gupta and Gupta 2016; Schiavon and Pilon-Smits 2017). Selenium accumulation between 407 and 769 ppm dry weight in shoots is associated with decreased plant size and leaf surface area in *B. juncea* (Bañuelos *et al.* 1997), suggesting that excess selenium slows growth. Specifically, we addressed the following questions: Do varieties of *B. juncea* differ in selenium uptake? At what tissue concentrations does selenium reduce leaf number and/or reproductive investment? And at what tissue concentrations does selenium reduce herbivory by *P. rapae*? To answer these questions, we grew plants in a growth chamber, watered half with a selenium solution and consequently measured size traits in the plants. We also conducted feeding trials in Petri dishes with individual *P. rapae* larvae. After the feeding trials, we measured selenium concentrations in the leaf used in the trial and in the larva to quantify the relationship between selenium accumulation and herbivory.

## Materials and Methods

### Growing conditions

We selected four commercial varieties of *B. juncea* (W. Atlee Burpee and Co.; www.burpee.com); Florida Broad Leaf, Mizuna, Southern Giant Curled and Tendergreen. Seeds were planted in 9-cm square pots with Metromix 360 potting medium (Sun Gro Horticulture; www.sungro.com) and grown in a growth chamber with 16 h days at 24 °C daytime temperature and 15.5 °C nighttime temperature. All plants were placed in flats with two plants of each variety and were watered from below by partially filling the tray. Flats were randomly located within the growth chamber. One week after germination, all varieties of *B. juncea* were fertilized with Osmocote Indoor and Outdoor Plus following the package instructions (The Scotts Company; www.osmocotegarden.com). Selenate treatments were begun 2 weeks after planting and continued every other day for 3 weeks. Fifteen plants of each variety were assigned to one of two treatments; a control treatment watered with tap water and a selenate treatment watered with 0.1 mM sodium selenate. Plants were watered by filling the flat with selenate solution or water; while this approach did not tightly regulate the amount of selenium each plant received, all plants were treated similarly.

### Data collection

Four weeks after planting, plant size was measured by counting total leaf number, measuring the height of the flowering stem, and counting flower number. None of the Mizuna plants and only half of the Tendergreen plants bolted during the study, resulting in a sample size of 15 plants per treatment and variety combination for leaf number, and 15 plants per treatment and variety combination in Southern Giant Curled and Florida Broad Leaf flowering stem height and flower number, with nine Tendergreen plants measured in the control treatment and eight measured in the selenate treatment for the reproductive traits. Due to increasing plant size, limited space in the growth chamber, and the absence of pollinators, the experiment was terminated before plants set seed.

*Pieris rapae* eggs were obtained from Carolina Biological Supply Company (www.carolina.com) and raised for 10–12 days on untreated plants of each variety in a separate location. When the plants were 6 weeks old, we performed individual feeding trials with the larvae in 100 m × 15 mm Petri dishes. A single leaf was cut from each plant and separated into halves along the midvein; half the leaf was placed in a Petri dish and half was dried for selenium analysis. Larvae were selected from food plants of the same variety whenever possible and added to the dish. The dish also contained a small piece of moist paper towel to increase humidity. Leaves and larvae were weighed at the beginning and end of the trial. We conducted three feeding trials, and each trial lasted 17–24 h. In total, 11 plants from each variety and treatment combination were assessed in the feeding trials. The larvae were frozen immediately after the trial for selenium analysis. We conducted a separate trial with 10 leaves to determine leaf weight loss due to wilting over a 24-h period. Leaves in Petri dishes with a paper towel but no larva lost an average of 29 % of their weight, and there was no difference between control and selenium-treated plants (*t* = 0.735, *df* = 8, *P* = 0.242).

We used an acid digestion and Inductively Coupled Plasma Optical Emission Spectrometry to measure selenium concentration in leaves and larvae. Leaves were dried prior to digestion and larvae were frozen. We digested leaves and larvae in a 2:1 solution of nitric acid:perchloric acid (Twyman 2005) and filtered this solution through a 1-μm filter. All samples were then analysed for selenium at 185, 196 and 204 nm, and we used standard curves generated from standards of known selenium concentration (High-Purity Standards; www.highpuritystandards.com). We averaged the values from each of the three wavelengths, and then calculated the leaf and larval concentrations of selenium in μg g^−1^ of tissue. The detectable threshold for selenium in our samples was 1 μg g^−1^.

### Statistical analysis

All statistical analyses were conducted in R (R Core Team 2016). Initial analyses showed no difference between flats and feeding trials and these variables were not included in further analyses. We compared selenium levels across varieties using a one-way ANOVA. We used two-way ANOVA to compare leaf number, height of flowering stem and flower number among varieties and between treatments. None of the Mizuna plants and only half of the Tendergreen plants bolted during the study, and Mizuna was not included in the analysis of flowering stem height or flower number. We compared the height of the flowering stem between treatments in Florida Broad Leaf with a *t*-test. To determine the relationship between selenium concentration and measures of growth, we used a linear model (lm in R) with variety and selenium concentration as covariates and determined the significance of selenium concentration on growth after variation due to variety had been accounted for. Only plants in the selenate treatment were included in these analyses.

We calculated the percent change in larva and leaf weight by subtracting the final weight from the initial weight and dividing by the initial weight. Percent change in leaf weight was normalized by changing the numbers to positive values and using a square root transformation. Percent change in larva weight was normalized by adding 40 to make all values positive and then taking the square root of this value. We used a two-way ANOVA to compare leaf and larva change in weight across varieties and between treatments. Using just the plants in the selenate treatment, we conducted a linear regression to determine whether leaf selenium concentrations determined the percent change in leaf weight and the percent change in larva weight. We also used linear regression to determine the relationship between the percent change in leaf weight and flower number in selenium-treated Florida Broad Leaf plants, the variety that showed the greatest accumulation of selenium and significant flower production.

## Results

**Figure 1.**
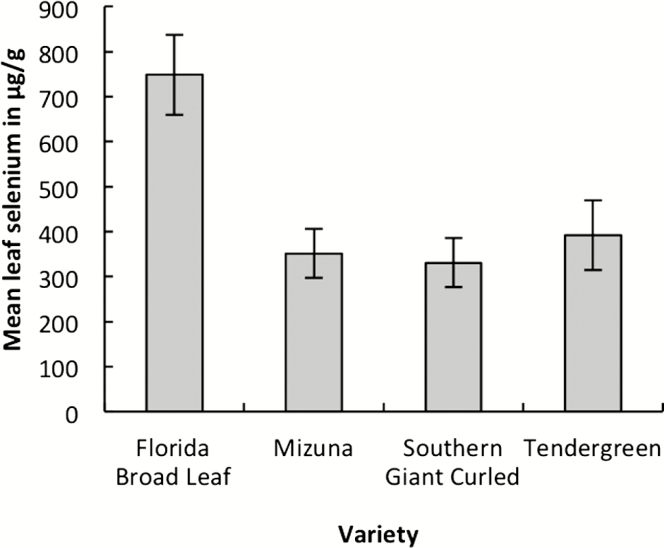
Selenium concentration in leaf tissue from four varieties of *Brassica juncea* watered with a 0.1 mM solution of sodium selenate. *N* = 10 per variety. Error bars are ±1 SE.

None of the plants in the control treatment had detectable levels of selenium in leaf tissue, while all of the plants in the selenate treatment did. Selenium uptake did differ by variety; leaf selenium concentration in Florida Broad Leaf was almost twice that found in the other three varieties (Fig. 1; *F*_3, 37_ = 7.82, *P* = 0.0004).

**Figure 2.**
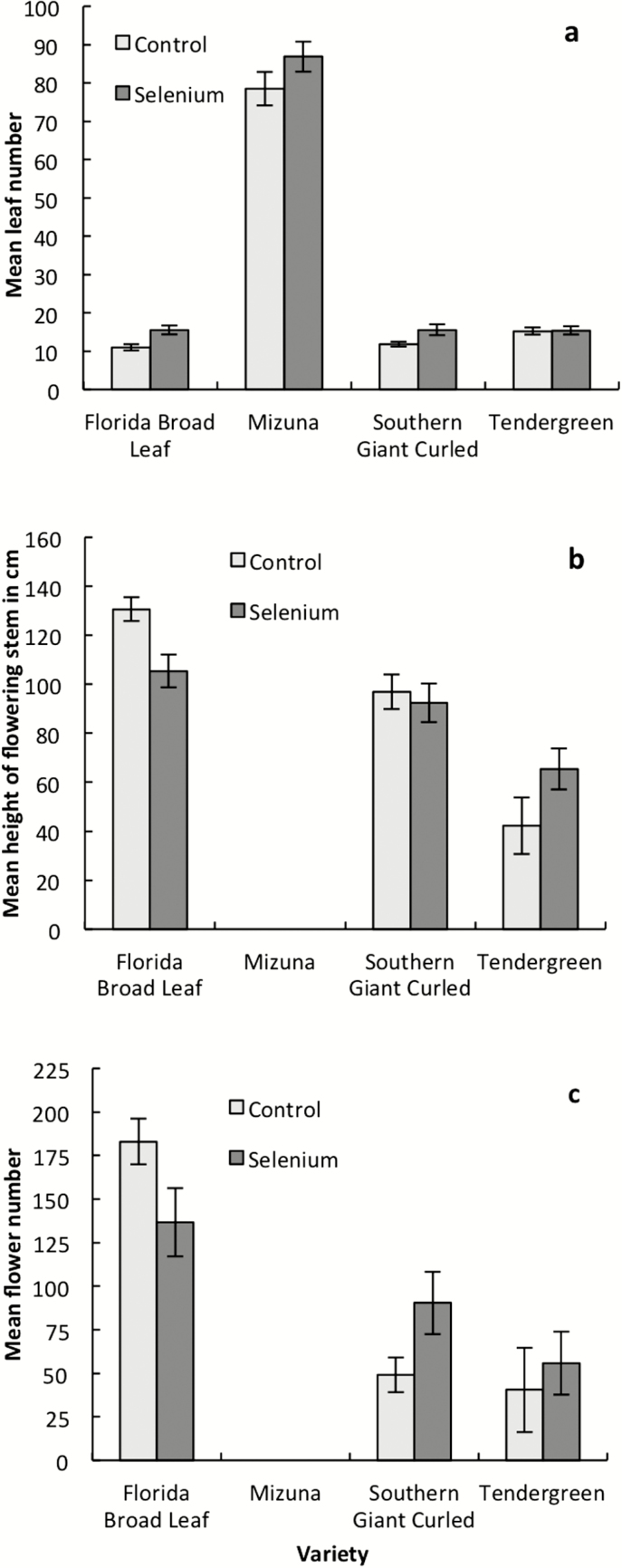
Leaf number (A), flowering stem height (B) and flower number (C) in four varieties of *Brassica juncea* either watered with 0.1 mM sodium selenate or in a control treatment. Mizuna did not flower during the study. Error bars are ±1 SE. See Table 1 for statistical results.

**Figure 3.**
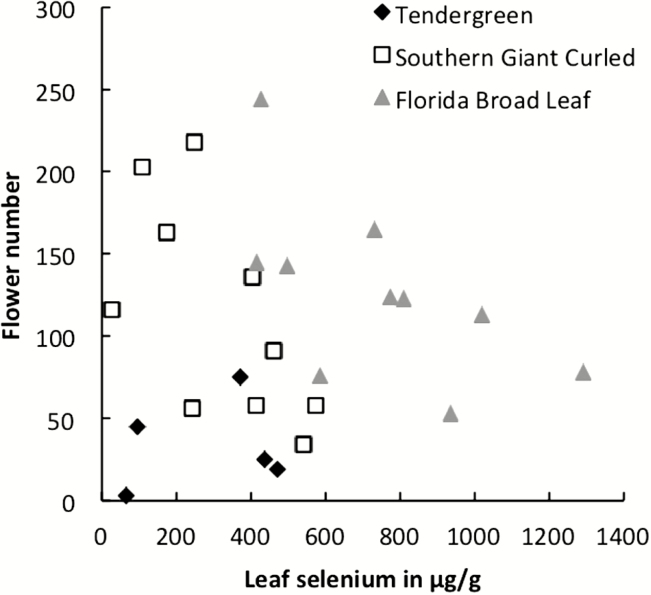
Leaf selenium concentration as a predictor of flower number in three varieties of *Brassica juncea* (diamonds, Tendergreen; squares, Southern Giant Curled; triangles, Florida Broad Leaf) watered with a 0.1 mM solution of sodium selenate.

The selenate treatment had little overall impact on plant size, and leaf number was slightly greater in plants watered with sodium selenate (Fig. 2; Table 1). The selenate treatment had no main effect on flower number or the height of the flowering stem, but the interaction between treatment and variety is significant, indicating that varieties had differing responses to the treatment. Within Florida Broad Leaf plants, the selenate treatment slightly decreased the height of the flowering stem (Fig. 2B; *t* = 3.02, *df* = 28, *P* = 0.0053). In addition, flower number decreased with an increase in selenium concentration in leaves (Fig. 3; coefficient = −0.0063, *F*_1, 22_ = 5.59, *P* = 0.0273). For plants accumulating selenium, leaf selenium concentration showed no relationship with the height of the flowering stem (coefficient = −0.031, *F*_1, 22_ = 2.51, *P* = 0.127), and greater selenium concentrations were associated with a slight increase in leaf number (coefficient = 3.235, *F*_1, 79_ = 5.09, *P* = 0.0269). While overall growth is not negatively impacted by the selenate treatment, increasing selenium concentration in the leaf was associated with a decrease in flower number.

**Table 1.** Two-way ANOVA for leaf number, flowering stem height, and flower number in four varieties of *Brassica juncea* receiving either a sodium selenate treatment or a control treatment, and percent weight change in leaves and larvae following feeding trials with *Pieris rapae* and leaves from these treatments. Flower number and percent change variables were square root transformed.

Source	*df*	Sum of squares	*F*	*P*-value
Leaf number				
Variety	3	105 583	454.45	<0.0001
Treatment	1	539	6.965	0.0095
Variety × treatment	3	249	1.071	0.3644
Error	111	8596		
Height of flowering stem				
Variety	2	45 673	31.296	<0.0001
Treatment	1	811	1.111	0.2950
Variety × treatment	2	6396	4.382	0.0160
Error	71	51 808		
Flower number				
Variety	2	580.3	21.23	<0.0001
Treatment	1	5.4	0.396	0.5313
Variety × treatment	2	101.0	3.695	0.0298
Error	70	956.5		
Percent change in leaf weight				
Variety	3	19.09	2.78	0.0464
Treatment	1	76.92	33.62	<0.0001
Variety × treatment	3	2.14	0.312	0.8170
Error	79	180.77		
Percent change in larva weight				
Variety	3	43.38	4.58	0.0052
Treatment	1	55.05	17.4	<0.0001
Variety × treatment	3	0.15	0.0159	0.9973
Error	77	243.13		

**Figure 4.**
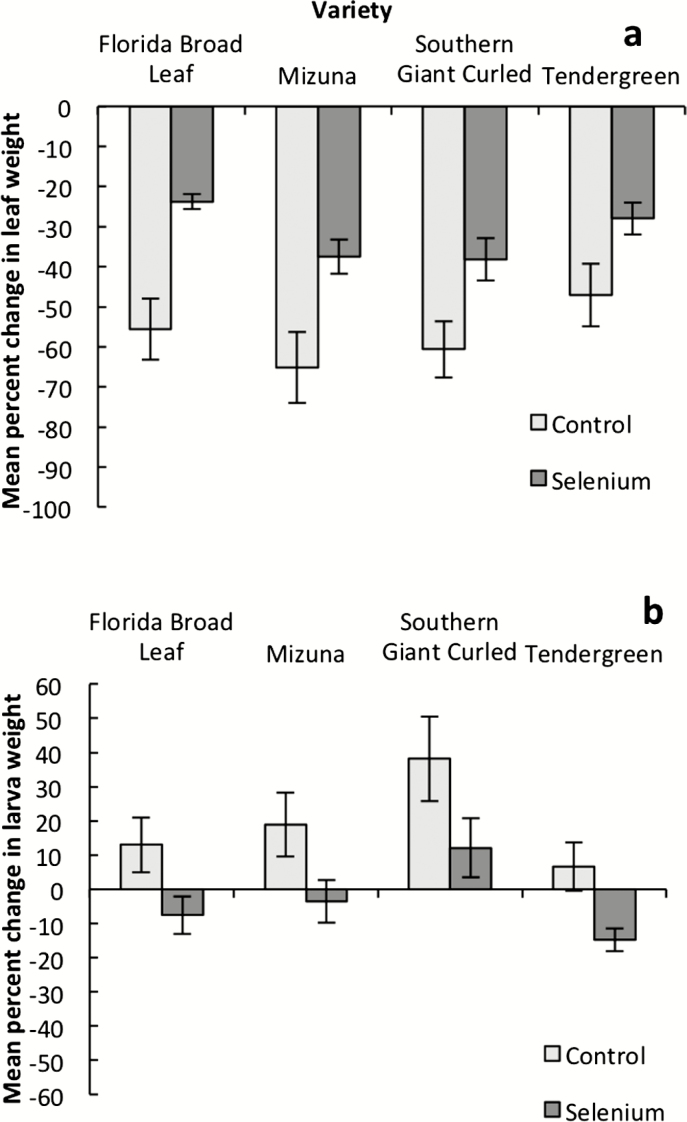
Mean percent change in leaf weight (A) and larva weight (B) for *Brassica juncea* leaves treated with a selenium or control treatment and fed to *Pieris rapae* larvae over a 17- to 24-h period. On average, 29 % of weight loss in leaves is due to wilting. Error bars are ±1 SE. See Table 1 for statistical results.

**Figure 5.**
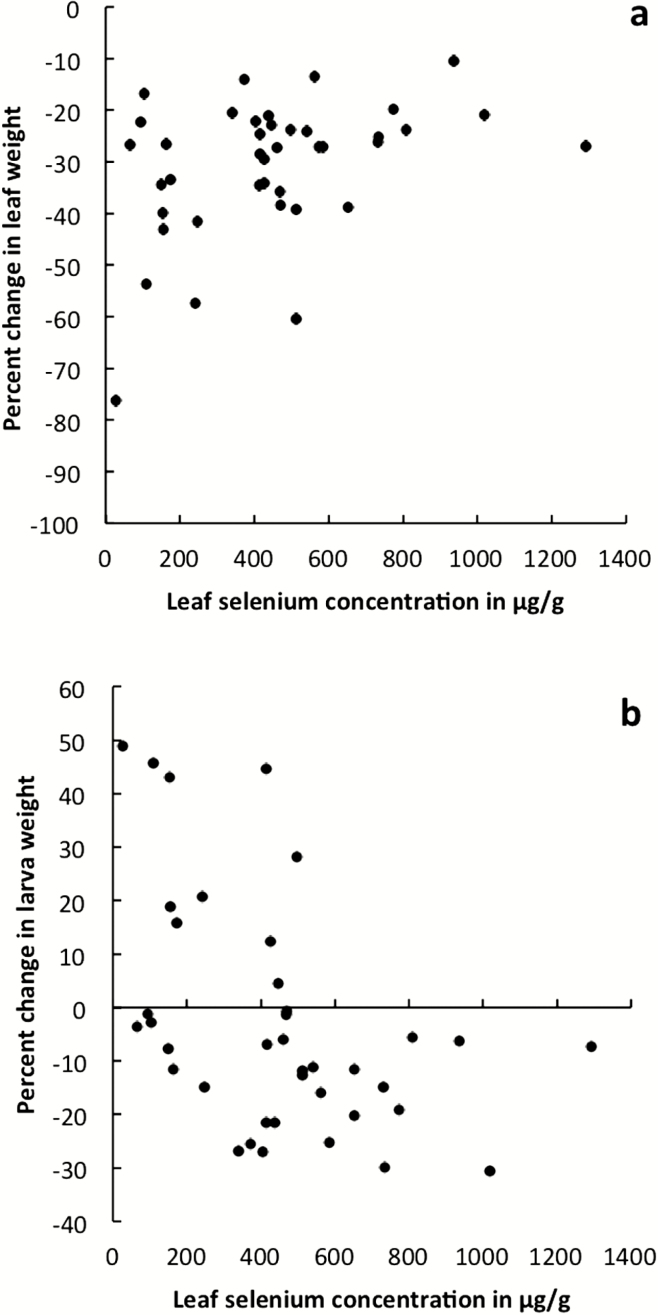
The relationship between leaf selenium concentration and percent change in leaf weight (A) and percent change in larva weight (B) for larvae placed with leaves from plants in the selenate treatment for a feeding trial.

The selenate treatment significantly decreased larval feeding in all varieties. Leaves from the selenate treatment lost considerably less biomass to herbivory than leaves from the control treatment (Fig. 4A; Table 1). In addition, the mean weight gain for larvae feeding on leaves from control plants was positive for all varieties, while larvae feeding on selenium-watered plants either gained no weight or lost weight on average (Fig. 4B; Table 1). Greater concentrations of selenium in leaf tissue significantly decreased herbivory; leaves with lower selenium concentrations lost more leaf tissue to herbivory (Fig. 5A; *R*^2^ = 0.132, *F*_1, 38_ = 6.95, *P* = 0.0121), and larvae gained more weight when feeding on leaves with lower selenium concentrations (Fig. 5B; *R*^2^ = 0.191, *F*_1, 37_ = 9.99, *P* = 0.0031). In Florida Broad Leaf, leaves from plants with lower flower number also lost less leaf tissue to herbivory in feeding trials (*R*^2^ = 0.363, *F*_1, 8_ = 6.14, *P* = 0.0383).

Only 41 % of larvae given leaves from the selenate treatment contained detectable levels of selenium at the end of the trial, suggesting that little or no leaf tissue was consumed by the majority of larvae given selenium-rich leaves. In larvae with detectable levels of selenium, the mean selenium concentration was 9.57 μg g^−1^, with a range from 1.17 to 29.13 μg g^−1^.

## Discussion

We found significant variation in selenium tissue concentrations within and between varieties of *B. juncea*, and keeping with the elemental defence hypothesis, we found that selenate decreased herbivory by a specialist herbivore. When given leaves with more than 500 μg g^−1^ selenium, larvae consistently lost weight and leaf consumption was low. However, plants with selenium concentrations above 500 μg g^−1^ also showed decreased flower number. In addition, the negative correlation between flower number and defence against herbivory in the variety with the highest selenium concentrations points to a possible trade-off between reproduction and defence. This balance between defence and toxicity may favour the maintenance of intermediate levels of selenium concentration in accumulators.

Considerable variation for selenium uptake was present among and within varieties in our study, and similar variation has been observed in other selenium accumulators. A population of *Symphyotrichum ericoides* growing on seleniferous soil showed much greater selenium uptake than a population of the same species growing on non-seleniferous soil, and at least some of this variation is attributable to ecotypic differences, as demonstrated by common-garden experiments (El Mehdawi *et al.* 2015). Sixteen populations of the selenium hyperaccumulator *S. pinnata* also showed variation in selenium uptake in a greenhouse study (Feist and Parker 2001). These findings suggest local adaptation to selenium-rich soils and a genetic basis for rates of element uptake.

The selenate treatment did potentially increase plant size in *B. juncea* at lower concentrations, as evidenced by the increased leaf number in the selenate treatment for some varieties. However, increasing concentrations of selenium in the leaf was associated with a decrease in flower number in the same plant (Fig. 3). Selenium may contribute to increased plant growth because of selenium-induced stress responses (Gupta and Gupta 2017) or the antioxidant effects of selenium at lower doses (Poschenrieder *et al.* 2013). The increase in leaf number may also represent a shift in biomass allocation within the plant in the presence of selenium rather than an overall growth increase. Prins *et al.* (2011) found that concentrations of selenium comparable to those in this study showed no effect on growth in *B. juncea*, but at higher concentrations of selenium biomass, seed weight and seed germination declined. Overall, selenate in the growing medium had a minor effect on plant growth at the concentrations in this study, but also had negative effects on flower production in plants that accumulated selenium to higher concentrations.

Selenium is potentially both a deterrent and a toxin for *P. rapae* feeding on *B. juncea*. Increasing leaf selenium concentration resulted in decreasing leaf consumption and larval weight loss, and this relationship was significant despite variability in the data due to larval behaviour and leaf wilting. The selenate treatment may have also induced other physiological changes in the plants that decreased feeding through other mechanisms. In larvae that ingested selenium, the mean selenium concentration was 9.57 μg g^−1^. Twelve micrograms per gram of selenium was lethal for crickets ingesting selenium-rich plant material (Freeman *et al.* 2007), suggesting that some larvae in our experiment ingested toxic levels of selenium, although selenium may be preferentially excreted in frass (Hanson *et al.* 2003). However, 59 % of the larvae exposed to selenium in leaves ingested no detectable level of selenium, indicating that the effects of the selenate treatment can also serve as a deterrent to feeding. Larvae may have detected the presence of high levels of selenium through contact chemosensation and stopped eating (Pentzold *et al.* 2017), and/or selenium volatilized by leaves may be detectable before feeding begins (Schiavon and Pilon-Smits 2017).

While *P. rapae* appears to be sensitive to relatively low selenium concentrations, herbivores vary in selenium tolerance across species. Spider mites can survive on plants with elevated levels of selenium (Quinn *et al.* 2010), and a population of diamondback moth larvae feeding on plants growing in seleniferous soil showed an increased ability to tolerate selenium in comparison to a population from a non-seleniferous site (Freeman *et al.* 2006). Plants that hyperaccumulate selenium to very high levels are still palatable to Se-resistant herbivores (Galeas *et al.* 2008; El Mehdawi and Pilon-Smits 2012; Pilon-Smits 2015), and two species of seed herbivores showed a tolerance for selenium when feeding on seeds from selenium hyperaccumulators (Freeman *et al.* 2012). A species of *Astragalus* that hyperaccumulates selenium is reported to be host to two moth species that appear to be tolerant to selenium and accumulate it in their own tissues (Valdez Barillas *et al.* 2012).

Given the evidence in this study and others for the ability of relatively low concentrations of selenium to serve as a deterrent and a toxin to herbivores, hyperaccumulators of selenium appear to represent an extreme phenotype. Very high levels of selenium may be a product of co-evolution with specialist herbivores that are also evolving tolerance to selenium (Boyd 2009; Cappa and Pilon-Smits 2014). Strong selection in an accumulator could favour molecular changes that upregulate and modify the sulfur assimilation pathway, and lead to specialized selenium assimilation pathways and storage (Pilon-Smits 2015; Schiavon and Pilon-Smits 2017). Hyperaccumulation may only evolve in environments where specialist herbivores consistently feed on plants that have consistent and abundant access to selenium in the soil, and lower levels of selenium may suffice as a defence for species in more variable environments.

We have shown that moderate selenium accumulation in a plant species that lacks the specialization mechanisms of a hyperaccumulator may nonetheless provide a defence against herbivory before reaching toxic levels that significantly reduce growth or reproduction. Low levels of selenium accumulation have been reported in a few other species not recognized as accumulators, and elemental defences could be more widespread than previously recognized. White clover and lettuce both accumulate selenium to levels above 45 μg g^−1^ dry weight when grown on seleniferous soil (Fleming 1962). Wheat can also accumulate selenium to concentrations of 45 μg g^−1^ on sulfur-deficient soils containing sodium selenate (Li *et al.* 2008). Metal accumulation is also reported to confer protection from herbivory at lower concentrations. Levels of several heavy metals in artificial diet were toxic to diamondback moths at levels far below the defined hyperaccumulator threshold, and in the case of zinc, were within the concentrations considered normal for plants (Coleman *et al.* 2005; Cheruiyot *et al.* 2013). In addition, four plant species growing in a metal-rich grassland were protected against herbivory at tissue metal concentrations well below hyperaccumulator status (Ribeiro *et al.* 2017). Non-lethal levels of elemental defences may also act in combination with plant secondary compounds to enhance protection from herbivores and pathogens (Boyd 2007, 2012), and could be a particularly important defence against specialist herbivores that have the ability to disarm secondary compound defences. Elemental defences could be more widespread than previously recognized, and further research investigating elemental defence in plants growing in soils with moderate to low concentrations of metals or selenium is warranted.

## Supporting Information

The complete data set gathered in this study is available in the online version of the article as: SI_Steven_and_Culver_data.xlsx.

plz053_suppl_Supplementary_Data

## Sources of Funding

This work was supported by Christopher Newport University.

## Contributions by the Authors

A.C. designed the experiment and collected data on plant growth and herbivory, A.C. and J.S. collected selenium concentration data, J.S. conducted data analysis, A.C. wrote the first draft of the manuscript and J.S. edited and expanded the manuscript.

## Conflict of Interest

None declared.
